# Synergic effects of oxygen supply and antioxidants on pancreatic β-cell spheroids

**DOI:** 10.1038/s41598-018-38011-6

**Published:** 2019-02-12

**Authors:** Dina Myasnikova, Tatsuya Osaki, Kisaki Onishi, Tatsuto Kageyama, Binbin Zhang Molino, Junji Fukuda

**Affiliations:** 1 Faculty of Engineering, Yokohama National University, 79-5 Tokiwadai, Hodogaya-ku, Yokohama, 240-8501 Japan; 2 Department of Mechanical Engineering, Massachusetts Institute of Technology, Cambridge, MA 02139 USA

## Abstract

Diabetes is one of the most common metabolic disorders, and is characterized by the inability to secrete/sense insulin and abnormal blood glucose concentration. Many researchers have concentrated their efforts on improving islet transplantation, in particular by fabricating bioartificial pancreatic islets *in vitro*. One of the critical points for the success of this research direction is the improvement of culture conditions, such as oxygen supply, in the engineering of bioartificial pancreatic islets to ensure their viability and functionality after transplantation. In this work, we fabricated microwell spheroid culture devices made of oxygen-permeable polydimethylsiloxane (PDMS), with which hypoxia in the core of bioartificial islets was alleviated and glucose-stimulated insulin secretion was increased ~2.5-fold compared to a device with the same configuration but made of non-oxygen-permeable plastic. We also demonstrated that antioxidants, such as ascorbic acid-2-phosphate (AA2P), could neutralize islet damage caused by increased reactive oxygen species (ROS) in the cell culture environment. These results suggest that supply of oxygen together with removal of ROS may lead to a better approach to prepare highly viable and functional bioartificial pancreatic islets.

## Introduction

According to the International Diabetes Federation, ~425 million people worldwide had diabetes in 2017, and the number of patients continues to increase over time^1^. There are two main types of diabetes, types 1 and 2. The mechanisms underlying each type are different, but both lead to abnormal glycemia and conditions that may require surgical intervention^2–4^. In the past, the only available surgical treatment for diabetes patients was whole pancreas transplantation, which was usually performed with kidney transplantation^5^, further complicating an already difficult procedure. Lately, islet-only transplantation has become possible, and is now considered to be more appropriate as it is less complicated and invasive^6^. Since the first successful islet transplantation by Ballinger and Lacy^6^, research in this area has focused on improving both the process of islet transplantation itself, and post- and pre-transplantation care. It has been shown that the current isolation processes and following culture conditions influence the expression levels of a wide range of genes^7^ in natural pancreatic islets, altering their functioning. Moreover, a shortage of suitable donors and post-transplantation problems connected with hypoxia and immune response^8^ limit the amount of available transplantable pancreatic islets. Thus, much effort has been made in the investigation of fabricating bioartificial islets (e.g., spheroids) from various cell sources, such as stem or iPS cells^8–10^.

Experimental and computational results have shown that both natural and bioartificial islets ≥150 µm in size experience core hypoxia during culture *in vitro* that leads to decreased cell viability and functionality^11^, resulting from the lack of extensive microvasculature naturally present for islets in the human body^12^. Pancreatic islets have a high demand for oxygen, considering the fact that they account for no more than 2% of pancreatic volume but receive ~10% of pancreatic blood flow^13^. Oxygen tension decreases in isolated islets and even mild hypoxia during culturing leads to impaired insulin secretion in response to glucose stimulation (GSIS)^14,15^. Thus, the key to successfully preparing natural pancreatic islets and fabricating bioartificial pancreatic islets is the provision of sufficient oxygen during culture.

Several approaches have been developed to address this issue. The most simple and direct method is to maintain pancreatic islets under hyperoxic conditions (35–50% oxygen)^16^. Although this approach alleviated core hypoxia, it only partially reduced islet mass loss. In another approach, oxygen tension in the culture medium was increased by placing polydimethylsiloxane (PDMS) rings with incorporated CaO_2_ that gradually generate oxygen under contact with culture medium^17^. This approach led to increased insulin secretion in monolayer culture of MIN6 cells. However, a potential drawback is that the oxygen release depends on the geometry of the PDMS insert and may create an oxygen gradient, exposing adjacent cells to a high oxygen tension, but less tension to cells located farther away. A third approach uses PDMS as an oxygen permeable material for fabrication of a spheroid culture device^12,18–22^. This type of device allows spatial separation of spheroids and provides uniform oxygen tension conditions. Moreover, compared to other spheroid fabrication approaches, such as the hanging drop technique^23^, this method allows more straightforward and large-scale preparation of spheroids. As shown previously in HepG2 and MIN6-m9 cell lines, improved oxygen supply reduced hypoxia and increased cell growth rate and functioning (as determined by albumin^12^ and insulin^18^ secretion for HepG2 and Min6-m9, respectively). However, excessive oxygen supply may lead to adverse effects and be harmful to cells because of accumulated reactive oxygen species (ROS)^11,24,25^.

Herein, we fabricated PDMS spheroid culture devices for preparation of MIN6 spheroids and investigated whether improved oxygen supply leads to reduced hypoxia in the core of the spheroids. ROS can be generated under high oxygen tension, and they accumulate in cells and potentially interfere with normal cell signaling; therefore, we explored the protective effects of antioxidants on pancreatic spheroids. Our approach may be beneficial for preparing bioartificial islets with improved viability and functionality for islet transplantation.

## Results and Discussion

### Characterization of pancreatic spheroids on oxygen permeable/impermeable spheroid culture devices

**Figure 1:**
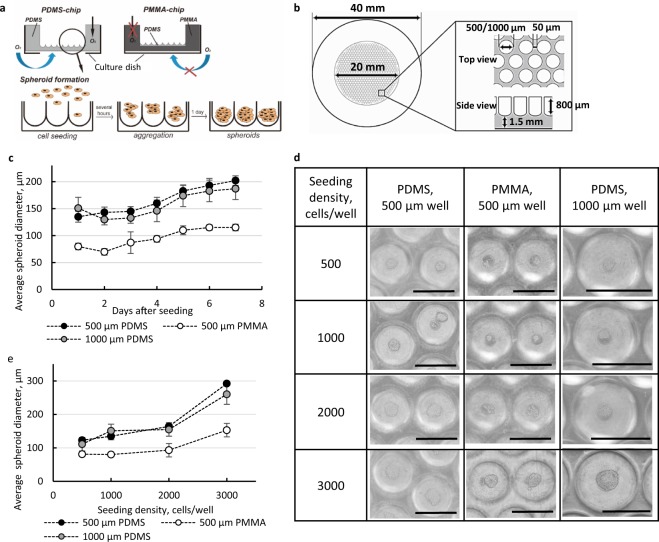
Optimization of spheroid fabrication conditions. (**a**) Schematic representation of PDMS and PMMA spheroid culture devices and the spheroid formation process. (**b**) Details of the spheroid culture device design. (**c,d,e**) Effects of well size and material of the device and seeding density of MIN6 cells. (**c**) Changes in average spheroid diameter (for representative seeding density of 1000 cells/well) in different devices during 7 days of culture (n = 10, *p* = 0.05). (**d**) Optical microscopy images of spheroids on Day 1 of culture. Scale bar of the first two columns: 500 µm; the third column: 1000 µm. (**e**) Average size of spheroids on Day 1 of culture (n = 10, *p* = 0.05).

To determine the feasibility of PDMS spheroid culture devices for the culture of pancreatic β-cells, we studied MIN6 and MIN6-m9 cells cultured either in the oxygen permeable devices made from PDMS (PDMS-chip) or in the devices with the same design but made of oxygen impermeable polymethylmethacrylate (PMMA-chip, Fig. 1a, Supplementary Fig. S1). Monolayer culture was also conducted with these cell lines as controls. Taking into account that natural pancreatic islets have an average size of 130 µm^26,27^ and consist of ~2500 cells^28^, we compared 2 designs of spheroid culture devices with well sizes of 500 and 1,000 µm (Fig. 1b) and seeding density in the range of 500–3,000 cells/well (Fig. 1c–e). The morphology of spheroids was nearly spherical for all tested conditions (Fig. 1d). At the lower cell seeding densities of 500 cells/well and 1,000 cells/well, the PDMS-chip of ⌀500 µm wells allowed formation of spheroids with diameters of 160 ± 7 µm and 180 ± 10 µm, respectively, while the same PMMA-chip produced spheroids with diameters of 100 ± 7 µm and 120 ± 6 µm, respectively (Fig. 1e). There was a difference of ~50 µm in the average size of spheroids between PMMA-chip and PDMS-chip at the first day of culture (Fig. 1c). Cell aggregation processes are connected with energy regeneration and thus may be closely associated with oxygen supply. Due to the proteolytic activity of trypsin, cell membrane proteins, including adhesive proteins such as members of the cadherin family, are often cleaved^29^. An increased amount of energy, thus more oxygen, is necessary to recover from trypsin damage, in particular for the regeneration of lost membrane molecules. In the present study, there was a difference in aggregation of cells between the two types of chips. In PMMA-chip, cells formed multiple small spheroids in some wells with single cells around, while in PDMS-chip almost all cells aggregated into a single spheroid in each well. Thus, we assumed that this difference is attributed to oxygen supply because it was the only difference between the two types of chips. Higher cell seeding densities in both 500-µm chips did not result in larger spheroids because not all the seeded cells were able to settle into wells and floating cells were aspirated during culture medium exchanges (Fig. 1e). The PDMS-chips with 1,000-µm wells allowed a size increase in spheroids and an increase of seeding densities from 500 cells/well to 3,000 cells/well due to more available space in which cells could settle.

To prove the benefits of using oxygen-permeable PDMS-chips for functioning of β-cells, we compared the morphology and insulin secretion ability of spheroids formed from 2 similar pancreatic β-cell lines MIN6^30^ and MIN6-m9^31^ cultured in PDMS- and PMMA-chips. According to the literature^31^, the MIN6 cell line is nonhomogeneous and consists of cells with different responses to glucose in terms of insulin secretion. Moreover, this cell line consists of cells positive for insulin and glucagon, indicating a mixture of β- and α-cells^32^. MIN6-m9, on the other hand, was cloned from one MIN6 cell with high response to glucose^31^ and is a cell line with homogeneous characteristics. Considering this fact, we also used the MIN6-m9 cell line in our experiments as a pure β-cell line. The culturing conditions for MIN6-m9 spheroids were the same as those for MIN6, as described in the Methods section.

**Figure 2:**
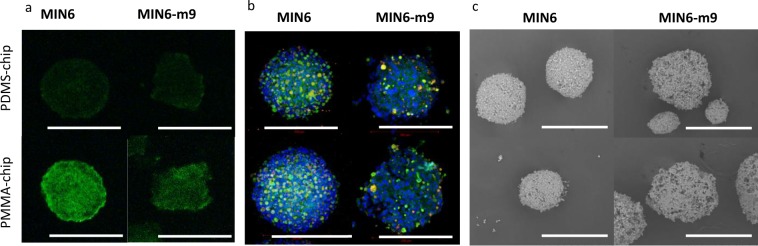
Qualitative characterization of culture spheroids. Immunofluorescent staining of MIN6 (left) and MIN6-m9 (right) spheroids (Day 4 after seeding) cultured in PDMS- (top) and PMMA-chips (bottom) for (**a**) HIF-1α. Staining was performed on 10 µm spheroid frozen sections. Scale bar: 200 µm. (**b**) Insulin (green), glucagon (red), and DAPI (blue). Staining was carried out for 3D spheroids. Scale bar: 200 µm. (**c**) SEM images of 3D spheroids treated with OsO_4_. Scale bar: 300 µm.

Both cell lines formed spheroids within 1 day in PMMA-/PDMS-chips. We investigated the hypoxia level in spheroids by fluorescent staining for hypoxia inducible factor (HIF)-1α 4 days after seeding. For both MIN6 and MIN6-m9, the fluorescence intensity (Fig. 2a) was 2-fold lower for spheroids cultured in PDMS-chips, indicating that oxygen-permeable devices induced decreased hypoxia levels in the spheroid structure. To investigate whether reduced hypoxia affected cell functioning, we first characterized spheroids by staining for insulin and glucagon (Fig. 2b); the latter is typically secreted by pancreatic α-cells, but was reported to be present in MIN6 cells as well^32^. Surprisingly, both MIN6 and MIN6-m9 spheroids had a small number of cells (~10%) positive both for insulin and glucagon. After confirming the double staining in spheroid cultures of both cell types, we found that even when cultured as a monolayer, there was a fraction of cells positive for glucagon (Supplementary Fig. S2). In the case of MIN6 cells, the presence of glucagon-positive cells can be explained by the initial composition of the cell line^32^. In the case of the MIN6-m9 cell line, which should be homogeneous in nature, a possible explanation is that the β-cells dedifferentiated to α-cells, which has been observed in natural pancreatic islets cultured *in vitro* in response to metabolic or oxidative stress^33^. Interestingly, in both types of spheroids, glucagon positive cells were concentrated on the periphery of spheroids similar to the natural structure of mouse islets^34^.

Scanning electron microscopy (SEM) was performed to examine spheroid structures (Fig. 2c). MIN6-m9 spheroids had a less dense structure with many gaps between cells, while MIN6 spheroids were well-defined and tightly packed with cells. This is likely due to the weaker cell-cell attachment between MIN6-m9 cells compared to MIN6 cells. Although further experiments are necessary to elucidate the mechanism, it may be associated with ability of the cells to secrete extracellular matrix or the strength of cell-cell adhesion^35^.

### Glucose-induced insulin synthesis and secretion

**Figure 3:**
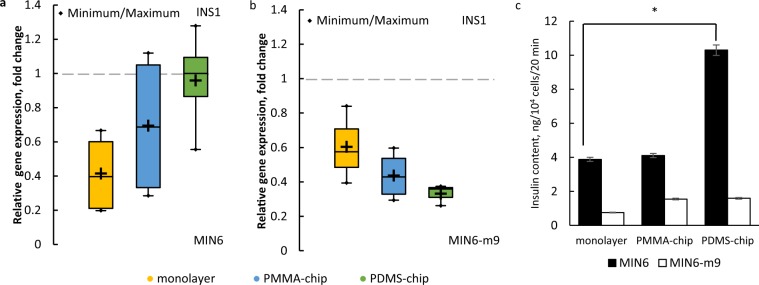
Quantitative comparison of cultured spheroids and monolayers. RT-PCR results for *INS1* expression in MIN6 (**a**) and MIN6-m9 (**b**) cultured for 4 days, represented as a relative fold change compared to the cells before seeding (horizontal line); ^+^ represents mean, n = 4, *p* = 0.05. (**c**) ELISA results for insulin secretion rate for MIN6 and MIN6-m9 cultured for 4 days, * represents statistically different results (n = 3, *p* = 0.05), error bars represent SE.

The most important characteristic of pancreatic islets, considering their function in the body, is the ability to secrete insulin in response to changing glucose concentration. Taking into consideration that deprived oxygen supply leads to impaired insulin secretion^15^, we hypothesized that improved oxygen supply *in vitro* would have a positive effect on the functionality of bioartificial pancreatic islets. To examine how different culture conditions influence cell characteristics, we compared expression of *INS1*, which encodes the precursor of insulin, and amounts of secreted insulin for MIN6 and MIN6-m9 cell lines cultured as monolayers or spheroids in different chips (Fig. 3). For MIN6 cells, *INS1* expression in spheroids was higher than that in monolayer culture, with the highest levels in spheroids cultured in oxygen-permeable PDMS-chips. These results are consistent with previous studies showing the importance of cell-cell contact for insulin secretion^36^. However, we observed strikingly different results for the MIN6-m9 cell line: spheroid culture with neither PMMA nor PDMS-chip led to improvement of *INS1* expression (Fig. 3b). The ELISA results were in accordance with the RT-PCR results; for the MIN6 cell line, we observed increasing amounts of secreted insulin in the order of monolayer <PMMA-chip <PDMS-chip, but for the MIN6-m9 cell line the order was the opposite. Because in the immunofluorescence staining images we observed weaker insulin staining in MIN6-m9 cells (Fig. 2b), we expected that both gene expression and insulin content would be less than that in MIN6 cells. Our results can be potentially explained by senescence of the cells^37^. Judging from these results, we decided to use only the MIN6 cell line for our subsequent experiments.

### Addition of antioxidants as scavengers of ROS

**Figure 4:**
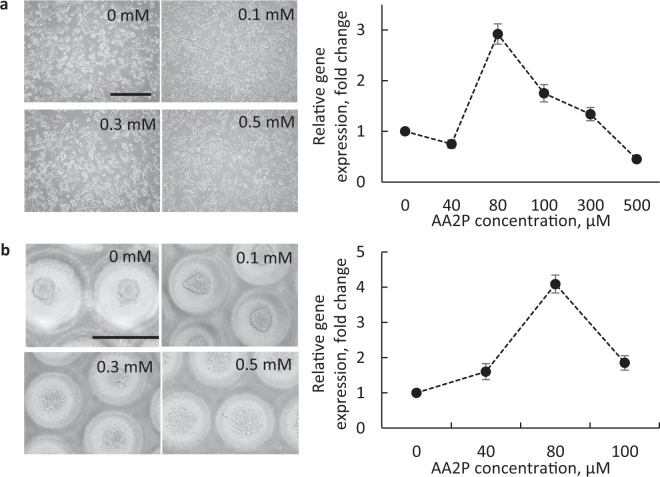
Influence of different concentrations of AA2P on morphology and *INS1* expression in MIN6 cells cultured in monolayer and spheroids. (**a**) Optical microscopy images of MIN6 monolayer cultured without and with addition of 0.1, 0.3, and 0.5 mM AA2P, and corresponding optimization of AA2P concentration in culture medium as determined by *INS1* expression. (**b**) Optical microscopy images of MIN6 spheroids cultured without and with addition of 0.1, 0.3, and 0.5 mM AA2P; and corresponding optimization of AA2P concentration in culture medium as determined by *INS1* expression. Scale bar: 500 μm.

Although the improved oxygen supply led to higher gene expression and insulin secretion, there was a concern regarding the level of ROS generated inside the cells. ROS appears to play an important role in insulin secretion^38–40^, and chronic exposure to high concentrations of ROS is damaging for cells and is known to play a role in β-cell dysfunction in diabetes^41^. In the latter case, secretion of antioxidants by the cells may disrupt the normal insulin-secreting mechanism^38^. We measured the amount of ROS-positive cells in spheroids cultured in different types of chips by flow cytometry. Spheroids cultured in the PDMS-chip had ~14% higher concentrations of intracellular ROS than those cultured in the PMMA-chip (Table 1). The elevated amount of ROS in the PDMS-chip may be associated with excessive oxygen concentration. We then studied the protective effects of antioxidants on β-cells from excessive ROS. Because L-ascorbic acid is unstable in water, we used its derivative ascorbic acid-2-phosphate (AA2P), which was reported to have the same antioxidant effect on fibroblasts as L-ascorbic acid^42,43^, but the effect lasted up to 1 month in the culture medium^42^. We determined the optimal concentration of AA2P in culture medium (Fig. 4). Concentrations >0.1 mM led to cell morphological changes including problems with cell attachment and death, which can be observed in the microscopy images (Fig. 4a,b). Lower concentrations had a positive effect on cell proliferation and *INS1* expression (Fig. 4a,b). We chose 0.08 mM to be the optimal concentration, as it induced the highest upregulation of *INS1* in both the monolayer and spheroid cultures and did not cause cell detachment or inability to form spheroids. It should be noted that in the case of spheroid culture, the toxic effect of AA2P at concentrations >0.1 mM was even more obvious than in monolayer culture; major problems with spheroid formation were observed at 0.3 mM, while at the same concentration in the monolayer culture the cells appeared to still not be damaged by the antioxidant.

**Table 1:** Relative amounts of live ROS positive (ROS+) and negative (ROS−) cells and median fluorescent intensity (MFI) of MIN6 cells cultured in different conditions, as assessed by flow cytometry of cells double-stained with DCFDA and 7-AAD viability dye. Unstained cells were used as a negative control. Cell treated with 0.1 mM H_2_O_2_ were used as a positive control. PDMS + AA2P = cells cultured in culture medium containing 0.08 mM AA2P. Similarly, PDMS + NAC = 5 mM NAC and PDMS + DTT = 0.1 mM DTT. PMMA and PDMS indicate cells cultured in PMMA- and PDMS-chips, respectively. All samples were taken on Day 4 of culture.

Sample	ROS−, %	ROS+, %	MFI
Negative control	99.95	0.05	4.86
Positive control	1.54	98.72	58.17
Monolayer	58.91	44.18	13.72
PMMA	56.82	50.12	15.01
PDMS	44.92	58.58	17.19
PDMS + AA2P	65.74	37.20	13.11
PDMS + DTT	62.33	40.83	13.11
PDMS + NAC	97.64	3.00	7.29

**Figure 5:**
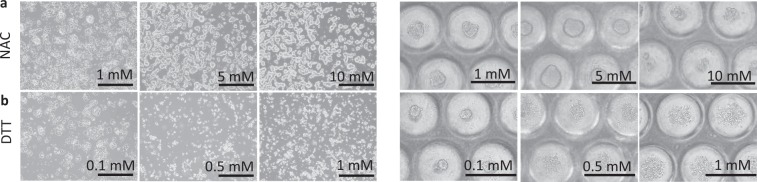
Influence of different concentrations of NAC and DTT on morphology of MIN6 monolayer and spheroids. Optical microscopy images of MIN6 monolayer (left) and spheroids (right) cultured with different concentrations of NAC and DTT. (**a**) 1, 5, and 10 mM NAC. (**b**) 0.1, 0.5, and 1 mM DTT. Scale bar: 500 μm.

To prove that this effect was due to the antioxidant and not vitamin activity of AA2P, we compared the effect with that of N-Acetyl-L-cysteine (NAC), which has been shown to have a protective effect on MIN6 monolayer cells against aldosterone-induced oxidative stress^44^, and with dithiothreitol (DTT), which has been shown to reduce cell apoptosis^45^. We tested several concentrations of NAC and DTT, based on concentrations used in previous studies^46^. As shown in the optical microscopy images (Fig. 5), both NAC and DTT had no negative effect on the morphology of cells either in monolayer or spheroid culture at 1–5 mM and 0.1 mM concentrations, respectively. 10 mM NAC did not have any negative effect on monolayer culture but led to an inability to form spheroids when cells were cultured in the PDMS-chip. DTT concentrations >0.1 mM led to changes in cell morphology, including their detachment and inability to form spheroids, similar to the effect observed for AA2P. Therefore, we decided to use NAC at 5 mM and DTT at 0.1 mM as controls for the flow cytometry experiments. All the antioxidants reduced ROS concentration and the lowest ROS concentration was observed with NAC addition. The latter may be connected with the higher concentrations of NAC comparing to other antioxidants (Table 1). The addition of AA2P led to a ~21% decrease in ROS concentration in the PDMS-chip. Addition of NAC led to the almost complete disappearance of ROS, while the effect of DTT was similar to that of AA2P (~18% decrease in ROS concentration). We concluded that AA2P, similar to NAC and DTT, scavenges ROS in cells and hence exhibited antioxidant activity in the tested culture conditions.

**Figure 6:**
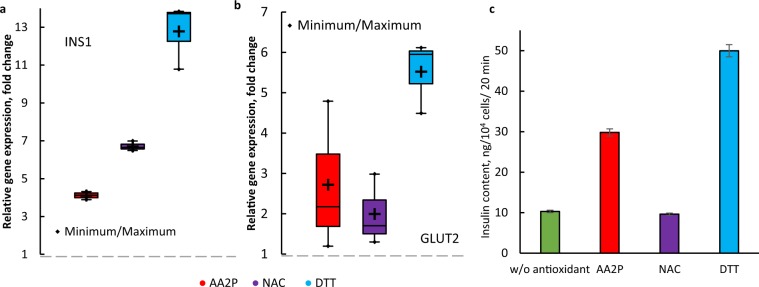
Comparison of the effect of different antioxidants added to culture medium. Influence of the optimum AA2P concentration on expression of *INS1* (**a**) and *GLUT2* (**b**) in MIN6 spheroids cultured in the PDMS-chip for 4 days, n = 4, *p* = 0.05. (**c**) Influence of antioxidants on insulin secretion rate of MIN6 spheroids cultured in the PDMS-chip for 4 days. ^+^ represents mean, error bars represent SE (n = 3, *p* = 0.05).

To show the influence of decreased ROS concentration on β-cell characteristics, we investigated the expression of *INS1* and glucose transporter type 2 (*GLUT2*) genes, and insulin secretion rate. All the antioxidants induced an increase in expression of both *INS1* and *GLUT2* (Fig. 6). The highest upregulation of both genes and increase in insulin secretion (~5-fold) were observed in the presence of DTT. However, the concentration of NAC that led to the largest decrease in ROS concentration did not increase insulin secretion, despite its upregulation of the selected genes. This may be due to the fact that although excessive amounts of ROS are harmful to cells, some ROS are still required for normal cell signaling. NAC may have been too effective as a scavenger of ROS and disrupted the normal functioning of cells^38^. The addition of AA2P showed a similar decrease in ROS concentration as that for DTT, but it led to an increase in insulin secretion by 3-fold compared to spheroids cultured in the PDMS-chip without AA2P. In this study, we showed that the PDMS-chip improved oxygen supply to pancreatic β-cell spheroids, decreasing hypoxia of cells in the core of spheroids and increasing insulin secretion in comparison to the PMMA-chip. However, rich oxygen conditions led to an increase in intracellular ROS concentration that could interfere with cell signaling and metabolism, including insulin secretion. Therefore, we examined neutralization of the negative effect of excessive amounts of ROS by addition of antioxidants and further demonstrated that addition of AA2P, as well as the addition of widely used antioxidants NAC and DTT, led to upregulation of *INS1* and *GLUT2* expression and increased insulin secretion in the spheroid culture of MIN6 pancreatic β-cells. It should be noted that we used a culture medium with 25 mM glucose, which is ~5-fold higher than normal blood glucose concentration for humans; thus, apart from excessive oxygen concentration, there was a possible risk of glucotoxicity in our system^47^. Elevated glucose concentrations can lead to formation and storage of increased amounts of advanced glycation products, which are known to be a source of ROS^41^. This aspect requires additional study, which we intend to perform in future experiments. Our results indicate that the design of cell culture conditions, as well as culture medium composition with respect to oxygen supply is essential when culturing pancreatic spheroids.

## Methods

### Preparation of oxygen-permeable spheroid culture devices

The 4 steps of oxygen-permeable PDMS-chip fabrication are as follows. (Supplementary Fig. S1). (1) The design was drawn using Adobe^®^ Illustrator software; the parameters of the mold were: diameter = 500/1,000 µm, interspace = 50 µm, well arrangement = triangular manner. The drilling pathway was created using VCarve Pro 6.5; depth of the wells = 800 µm. (2) The negative mold was fabricating by drilling into a circular piece (diameter = 40 mm, thickness = 2 mm) of polystyrene plastic using a milling machine (MODELA PROII MDX-540, Roland, Hamamatsu, Japan; Supplementary Fig. S1B) according to the design. (3) The replica mold was produced by pouring an epoxy resin solution consisting of 2:1 (w/w) of resin:hardener (Crystal Resin NEO, Nissen Chemical Corp., Tokyo, Japan) into the negative mold placed into a container with the same diameter (Supplementary Fig. S1c). The container with epoxy resin was left for 48 h at room temperature to cure, and then the replica mold was separated from the negative mold. (4) The PDMS-chip was fabricated by pouring PDMS solution consisting of 10:1 (w/w) prepolymer:catalyst (Shin-Etsu silicone, Shin-Etsu Chemical, Tokyo, Japan) into the replica mold, and deaerating it in a desiccator connected to a vacuum pump and heating for 30 min at 80 °C. The resultant chip was peeled from the replica mold, autoclaved, and treated with 4% Pluronic solution (Pluronic F-127, Sigma-Aldrich, Tokyo, Japan) in Milli-Q water to prevent cell attachment during culturing.

### Preparation of the oxygen-impermeable spheroid culture device

The oxygen impermeable spheroid culture device was made from PMMA. The PMMA-chip consisted of 2 parts (Fig. 1): a PMMA backbone and a PDMS insert with wells. The PMMA backbone consisted of a wide ring (outside diameter = 53 mm; inside diameter = 40 mm) that was cut from a 1 cm-thick PMMA plate (Hazaiya, Tokyo, Japan); and a bottom part that was cut from a 2-mm-thick plate (Comnet Co., Ltd., Kobe, Japan, Japan) on a laser cutter (LaserPro, Comnet Co., Ltd.). These were glued with adhesive (Acrysunday Adhesive, Acrysunday Co., Ltd., Tokyo, Japan). The PDMS insert with wells was cut from the PDMS-chip made as described above, autoclaved, and inserted into the PMMA-chip. Next, it was treated with Pluronic solution to prevent cell attachment during culturing.

### Cell culture

Mouse insulinoma β-cell lines MIN6 (gift from Prof. Miyazaki, Osaka University, Osaka, Japan)^30^ and MIN6-m9 (gift from Prof. Seino, Kobe University, Kobe, Japan)^31^ were both cultured in DMEM (4.5 g/L glucose, Sigma-Aldrich) supplemented with 10% fetal bovine serum (Biowest, Funakoshi Co., Ltd, Tokyo Japan), 1% penicillin/streptomycin (Gibco, Thermo Fisher Scientific, Yokohama Japan), and 5 × 10^−4^% β-mercaptoethanol (Sigma-Aldrich). All cell cultures were incubated at 37 °C in 5% CO_2_. Culture medium was changed every 2 days.

Before seeding in spheroid culture devices and as a control, both cell types were cultured in monolayer and were passaged by trypsinization (0.25% Trypsin-EDTA, Gibco, Thermo Fisher Scientific) once a week with a split ratio of 1:4. Seeding cell density for both PDMS- and PMMA-chips was varied at a range from 500 to 3,000 cells/well during optimization, and 1,000 cells/well was chosen for further experiments.

For the experiment with antioxidants: L-Ascorbic Acid Phosphate Magnesium Salt n-Hydrate (AA2P, FUJIFILM Wako Laboratory Chemicals, Tokyo, Japan) concentration in culture medium was at a range of 0.04–0.5 mM; N-Acetyl-L-cysteine (NAC, Sigma-Aldrich) was at a range of 1–10 mM; and DTT (Funakoshi, Co., Ltd., Tokyo, Japan) was at a range of 0.1–1 mM.

### Immunofluorescence staining for HIF1α, insulin, and glucagon

Immunofluorescence staining was performed for MIN6 and MIN6-m9 3D spheroids and frozen sections of spheroids. For direct staining, spheroids were fixed with 4% paraformaldehyde in PBS solution (FUJIFILM Wako Laboratory Chemicals) for 30 min at 4 °C, then washed 3 times with PBS (Gibco, Thermo Fisher Scientific). After blocking with blocking buffer (10% BSA KPL Diluent/Blocking solution, Seracare, Milford, MA, USA) for 1 h, a mixture of primary antibodies for insulin (Insulin Antibody H-86, rabbit polyclonal, Santa Cruz Biotechnology, Dallas, TX, USA) and glucagon (Glucagon Antibody C-18, goat polyclonal, Santa Cruz Biotechnology) was added (1:2,000 dilution) to spheroids, and they were kept overnight at 4 °C. Next, spheroids were washed 3 times with 0.1% PBS-Tween 20 (Sigma-Aldrich), and secondary antibodies (Alexa Fluor 488 Donkey Anti-Rabbit IgG H&L and Alexa Fluor 555 Donkey Anti-Goat IgG H&L, Abcam, Tokyo, Japan) were added (1:400 dilution) and kept for 2 h at room temperature. After that, spheroids were washed 3 times with PBS, treated with DAPI (1:1,000 dilution; Sigma-Aldrich) for 10 min, washed with PBS 2 additional times and observed under a confocal microscope (LSM 700, ZEISS, Tokyo, Japan). In case of HIF1α staining, MIN6 and MIN6-m9 spheroids were stained with Hypoxyprobe-1 (Hypoxyprobe-1 OMNI Kit, CosmoBio Co., Ltd, Tokyo, Japan) according to manufacturer protocol. After that, spheroids were fixed as it is described below, and the frozen sections were observed under the confocal microscope.

For frozen sectioning, spheroids were fixed with 4% paraformaldehyde for 30 min at 4 °C, rinsed 3 times with PBS, successively submerged into 10%, 20%, and 30% sucrose solutions (FUJIFILM Wako Laboratory Chemicals) for 1 h respectively, at room temperature. After that, spheroids were transferred to a cryo-dish, the sucrose solution was carefully aspirated so that only spheroids would remain, and the cryo-dish was then filled with Tissue-Tek O.C.T. compound (Sakura Finetek, Tokyo, Japan). The resultant sample was snap-frozen with liquid nitrogen, and then kept at −80 °C until cutting. Sections were cut at 10 µm thickness using a cryostat microtome, placed on a glass slide, and then stained in the same way as described for 3D spheroids.

### SEM

For SEM images, the culture medium was aspirated from the PDMS-chip, and spheroids were washed 3X with PBS. They were then fixed with a mixture of 2.5% glutaraldehyde (FUJIFILM Wako Laboratory Chemicals) and 2% formaldehyde for 1 h at room temperature and washed with PBS one more time. Fixed spheroids were treated with 1% osmium tetroxide (OsO_4_) solution (Electron Microscopy Sciences, Hatfield, USA) in PBS for 1 h at 4 °C, washed with Milli-Q water and dehydrated by successive treatment with 30%, 50%, 70%, 90% ethanol solution (FUJIFILM Wako Laboratory Chemicals), for 5 min each, followed by washing 3X with 100% ethanol solution, 5 min each, at room temperature. Ethanol was exchanged with t-butanol by washing the spheroids 2X with 100% t-butanol (Sigma-Aldrich). The samples were first kept at 4 °C for 1 h, transferred to −30 °C for 1 h, and finally lyophilized in a freeze dryer overnight (pressure = 10 Pa, temperature = −45 °C). The SEM images were obtained from a Miniscope TM-1,000 SEM (Hitachi, Tokyo, Japan).

### RT-PCR

Total RNA was extracted from cultured monolayer/spheroids using the RNeasy kit (Qiagen, Tokyo, Japan) according to the manufacturer’s protocol. An additional step was added to break the spheroids before the extraction: spheroids were added to tubes with beads (BioMaster, Nippi, Japan), shaken for 5 s, and then total RNA was extracted according to the manufacturer’s protocol. On the day of extraction, recovered total RNA was reverse transcribed to cDNA using the ReverTra Ace qPCR RT Kit (TOYOBO Co., Ltd., Osaka, Japan), according to the manufacturer’s protocol. cDNA samples were kept at −80 °C. RT-PCR was performed on a StepOne Real-time PCR system (Applied Biosystems, Thermo Fisher Scientific), using SYBR Green Premix Ex Taq II (Clontech, Takara Bio, Tokyo, Japan). The following primers were acquired from Takara Bio: **EEF2** forward 5′-CATGTTTGTGGTCAAGGCATAC-3′ and reverse 5′-TTGTCAAAAGGATCCCCAGG-3′; **INS1** forward 5′-CCCTTAGTGACCAGCTATAATCAGAGA-3′ and reverse 5′-ACCACAAAGATGCTGTTTGACAA-3′; **GLUT2** forward 5′-TCAGAAGACAAGATCACCGGA-3′ and reverse 5′-GCTGGTGTGACTGTAAGTGGG-3′. Eukaryotic translation elongation factor 2 (EEF2) mRNA was used as the internal standard in all experiments. The RT-PCR experiment was repeated at least twice for cDNA prepared from 4 batches.

### ELISA

C-peptide ELISA was performed to assess the insulin secretion rate. First, cultured monolayers or spheroids were washed with PBS and treated with DMEM containing no glucose (Gibco, Thermo Fisher Scientific) for 2 h. After that, cells were washed with PBS and treated with DMEM containing 4.5 g/L glucose for 20 min. Aliquots of 500 µL were collected and frozen immediately for the assays. A mouse C-peptide ELISA kit (U-type, FUJIFILM Wako Shibayagi Corporation, Shibukawa, Japan) was used for ELISA. Measurements were made according to the manufacturer’s protocol. The optical absorption was measured by a plate reader at 450 nm (reference wavelength = 620 nm).

### Flow cytometry

Spheroids and monolayers were cultured for 4 days, and then washed with PBS and treated with 2′,7′-dichlorofluorescin diacetate dye (DCFDA, Sigma-Aldrich) for 1 h to stain ROS-positive cells. Monolayer cells were trypsinized and collected in PBS. Spheroids were washed with PBS, and then treated with Accutase (Innovative Cell Technologies, Thermo Fisher Scientific) for 20 min in an 37 °C incubator, centrifuged at 6,000 rpm for 30 s and resuspended in PBS. Before measuring the number of ROS-positive cells, all samples were treated with live/dead cell discriminator 7-AAD viability dye (Beckman Coulter, Tokyo, Japan). Cells were counted with a MoFLo Astrios flow cytometer (Beckman Coulter). We used unstained cells as a negative control and cells treated with 0.1 mM H_2_O_2_ as a positive control. Additional details are provided in Supplementary Fig. S3.

### Statistical analysis

Spheroid size measurements, insulin content, and *INS1* expression shown in Fig. 5 are expressed as the mean value ± SE, and one-way ANOVA with Tukey’s HSD test were performed. Tests were performed using XLSTAT software (Addinsoft SARL, New York, NY, USA). Gene expression is represented as box-and-whisker plots; the lower and upper limits of the boxes are the 1^st^ and 3^rd^ quartiles, and whiskers represent the minimum and maximum of all data.

1^st^ and 3^rd^ quartiles, and whiskers represent the minimum and maximum of all data.

## Supplementary information

Dataset 1

## Data Availability

Authors can confirm that all relevant data are included in the article and/or its supplementary information files.
