# Supplementary material for: Synergic effects of oxygen supply and antioxidants on pancreatic β-cell spheroids
Source: Sci Rep. 2019 Feb 12;9:1802. doi: 10.1038/s41598-018-38011-6 (PMC6372787; doi:10.1038/s41598-018-38011-6)
Supplement: Supplementary file 1 — Dataset 1 [file 41598_2018_38011_MOESM1_ESM.pdf]

Synergic effects of oxygen supply and antioxidants on pancreatic β-cell spheroids

Dina Myasnikova[1], Tatsuya Osaki[1, 2], Kisaki Onishi[1], Tatsuto Kageyama[1], Binbin Zhang Molino[1], Junji Fukuda[1*]

[1] Faculty of Engineering, Yokohama National University, 79-5 Tokiwadai, Hodogaya-ku, Yokohama 240-8501, Japan

[2] Department of Mechanical Engineering, Massachusetts Institute of Technology, Cambridge, MA, 02139, USA

*Corresponding author: Junji Fukuda (fukuda@ynu.ac.jp)

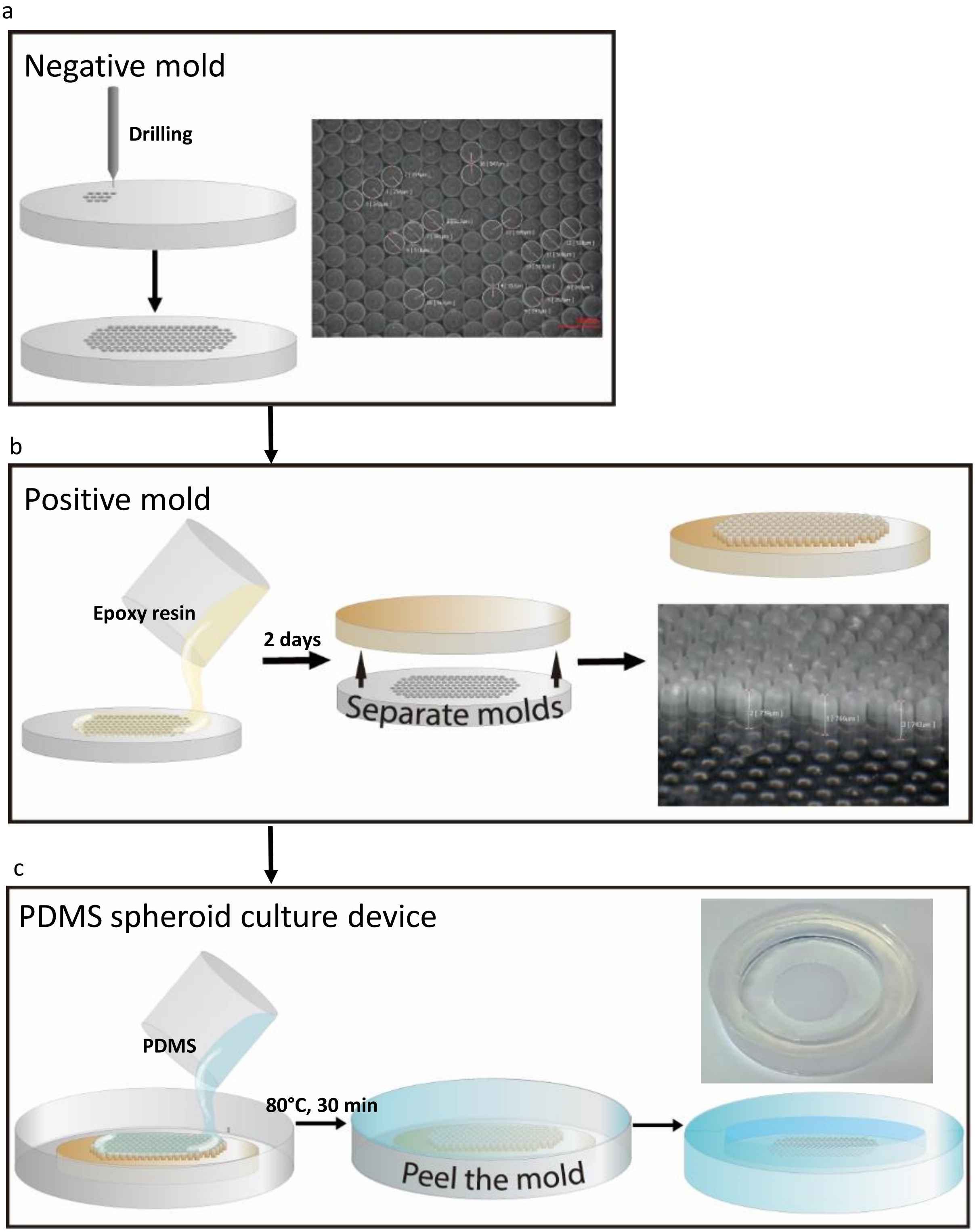

Fig.S1. Fabrication of PDMS spheroid plate. a: Fabrication of a negative mold by drilling wells in polystyrene circle and photo of the resulted mold. **b**: fabrication of a positive mold y pouring epoxy resin solution onto negative mold and the photo of the resulted mold. **c**: fabrication of PDMS-chip by pouring PDMS solution on positive mold and the photo of the final chip.

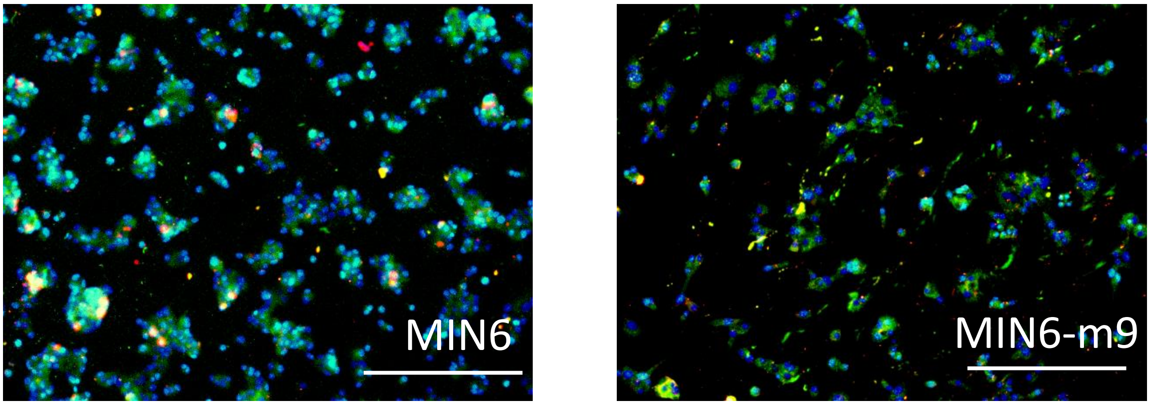

Fig.2S. Immunofluorescent staining of MIN6 (left) and MIN6-m9 (right) monolayers (day 2 after seeding) for insulin (green), glucagon (red) and DAPI (blue). Scale bar — 500 µm.

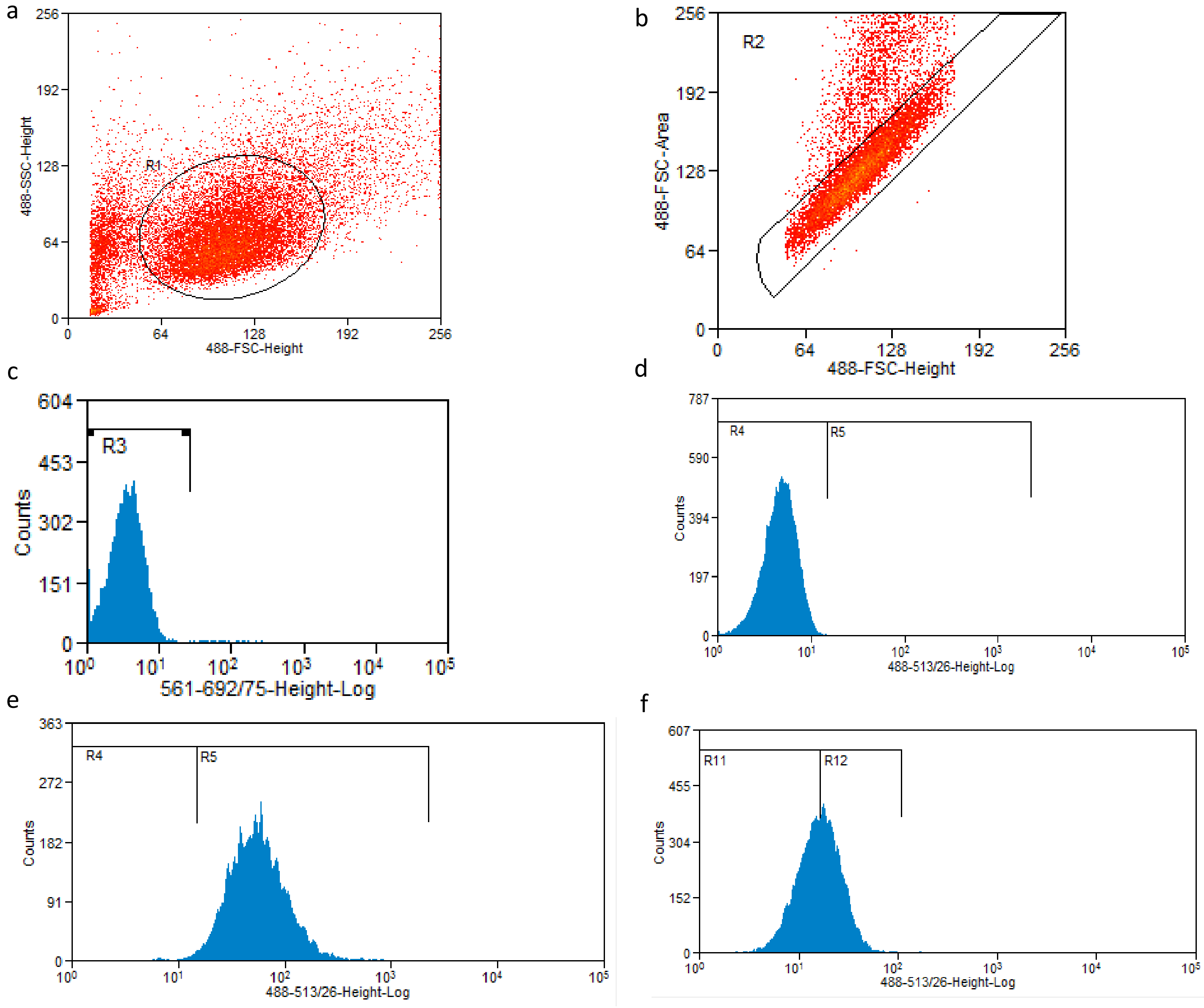

Fig.3S. The gating tree for flow cytometry experiment. Chosen laser line: 488 nm. Used emission filters: 692/75 for 7-AAD Viability Dye and 513/26 for DCFDA. **a**: FSC/SSC to **b**: SSC/pulse width to exclude events that could represent more than 1 cell to **c**: live gate to **d**: DCFDA negative to **e**: DCFDA positive. f: the example of a sample result with the set gates. A total of ~10,000 cells were analyzed for each sample.
